# Supplementary material for: A Comprehensive Pan-Cancer Analysis of the Regulation and Prognostic Effect of Coat Complex Subunit Zeta 1
Source: Genes (Basel). 2023 Apr 10;14(4):889. doi: 10.3390/genes14040889 (PMC10137353; doi:10.3390/genes14040889)
Supplement: Supplementary file 1 [file genes-14-00889-s001.zip › genes-2250660-supplementary.pdf]

Supplementary Materials

Figure S1. The expression level of COPZ1 in different kinds of cancer in the GEPIA database.

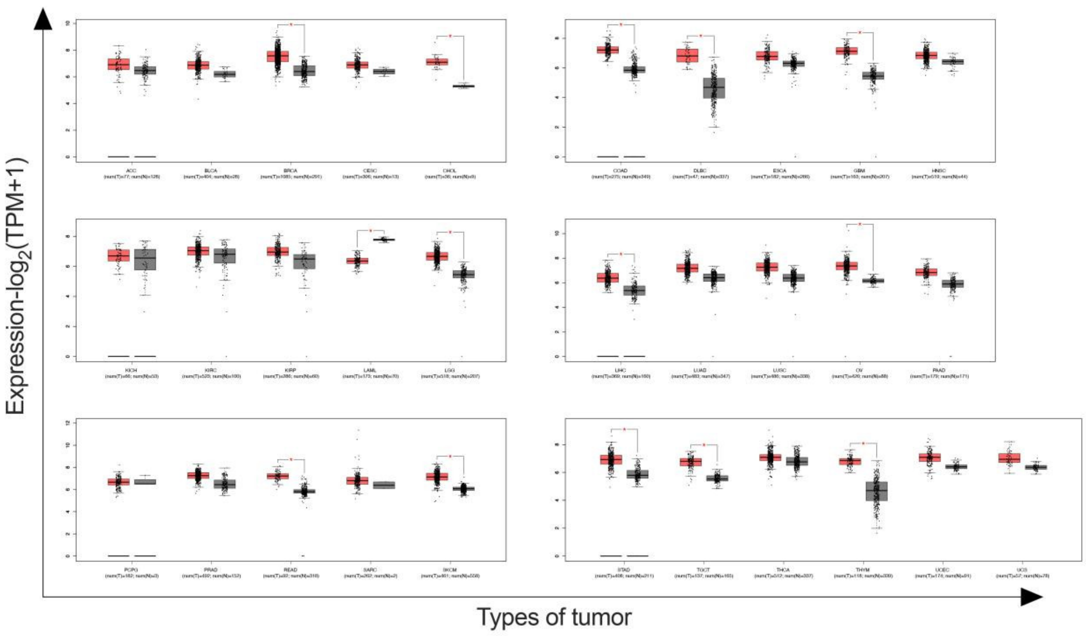

Figure S2. The expression level of COPZ1 in different kinds of COPZ1 CNV status among six types of cancer.

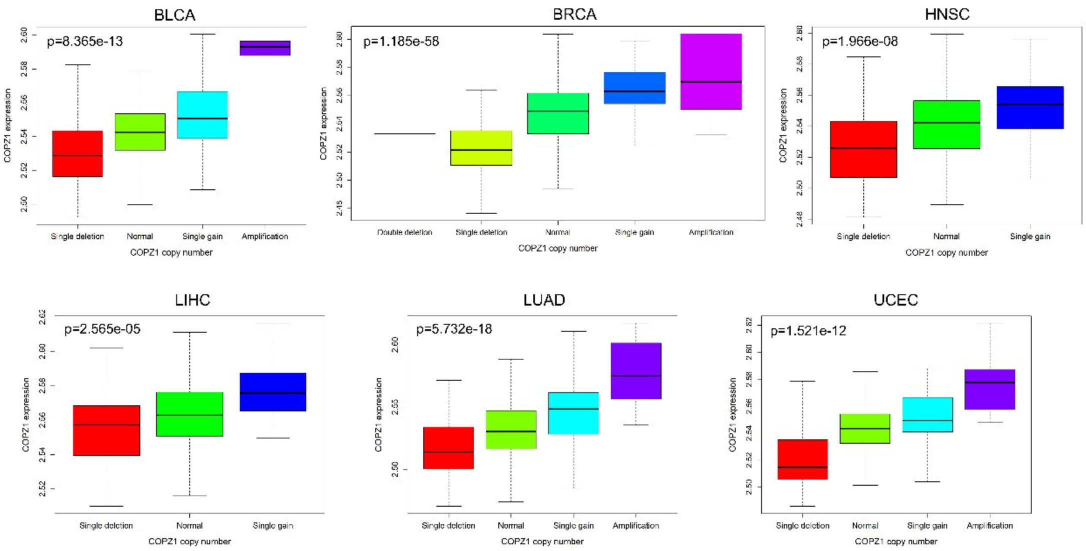

Figure S3. Correlation analysis between COPZ1 expression and upregulated transcription factors' expression in six types of cancer.

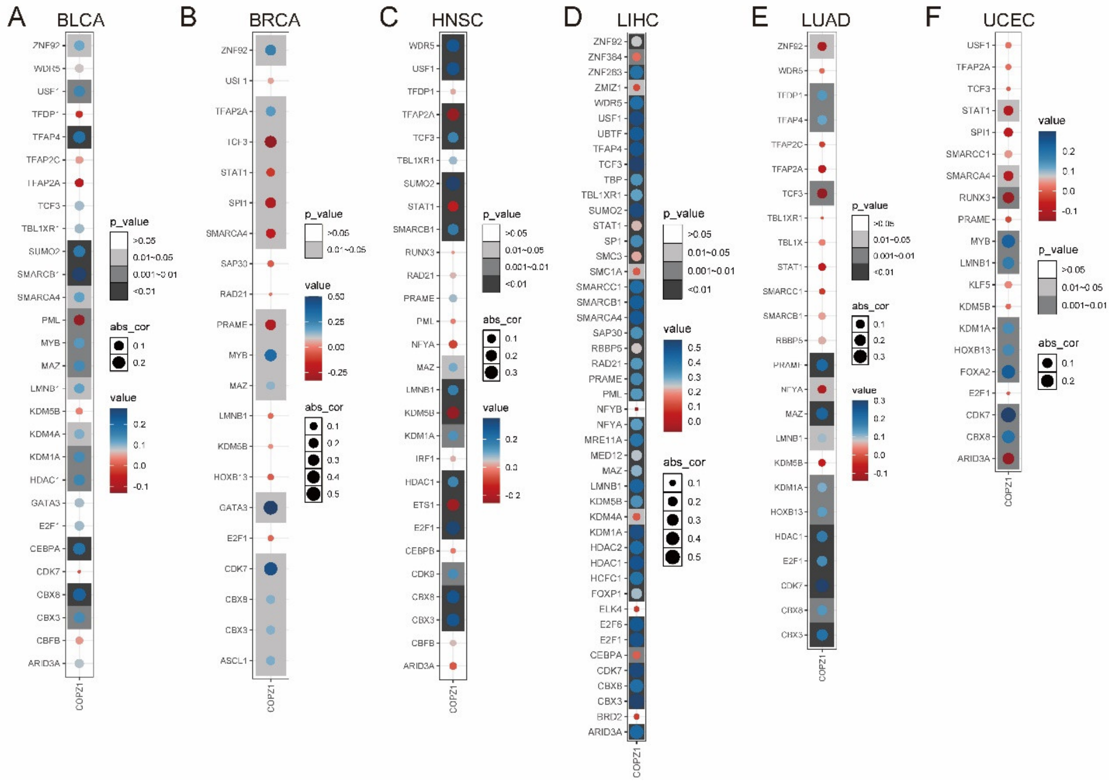

Figure S4. Correlation analysis between COPZ1 expression and down-regulated MicroRNAs' expression in six types of cancer.

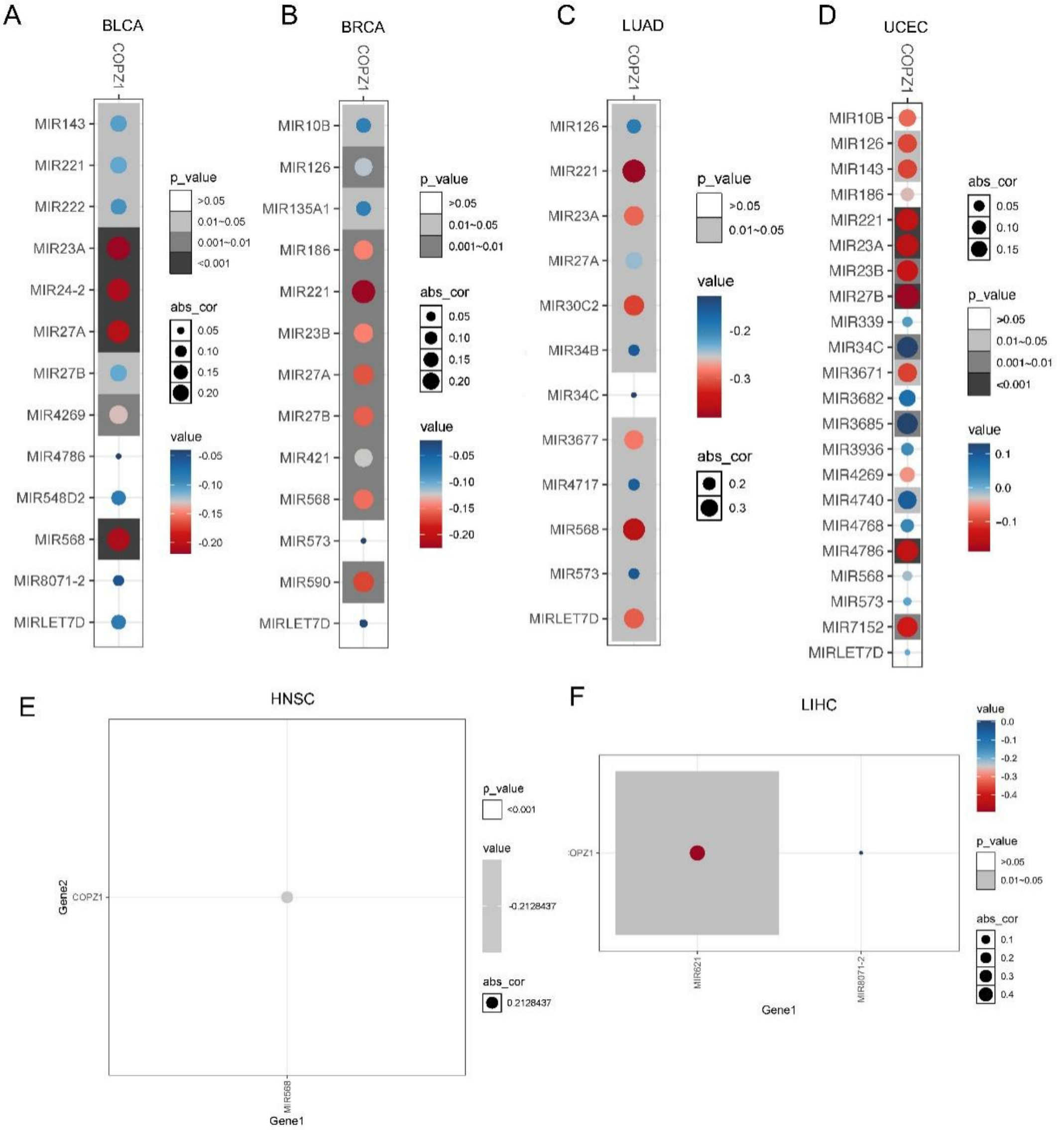

Figure S5. Correlation analysis between COPZ1 expression and TMB and MSI in multiple types of cancer.

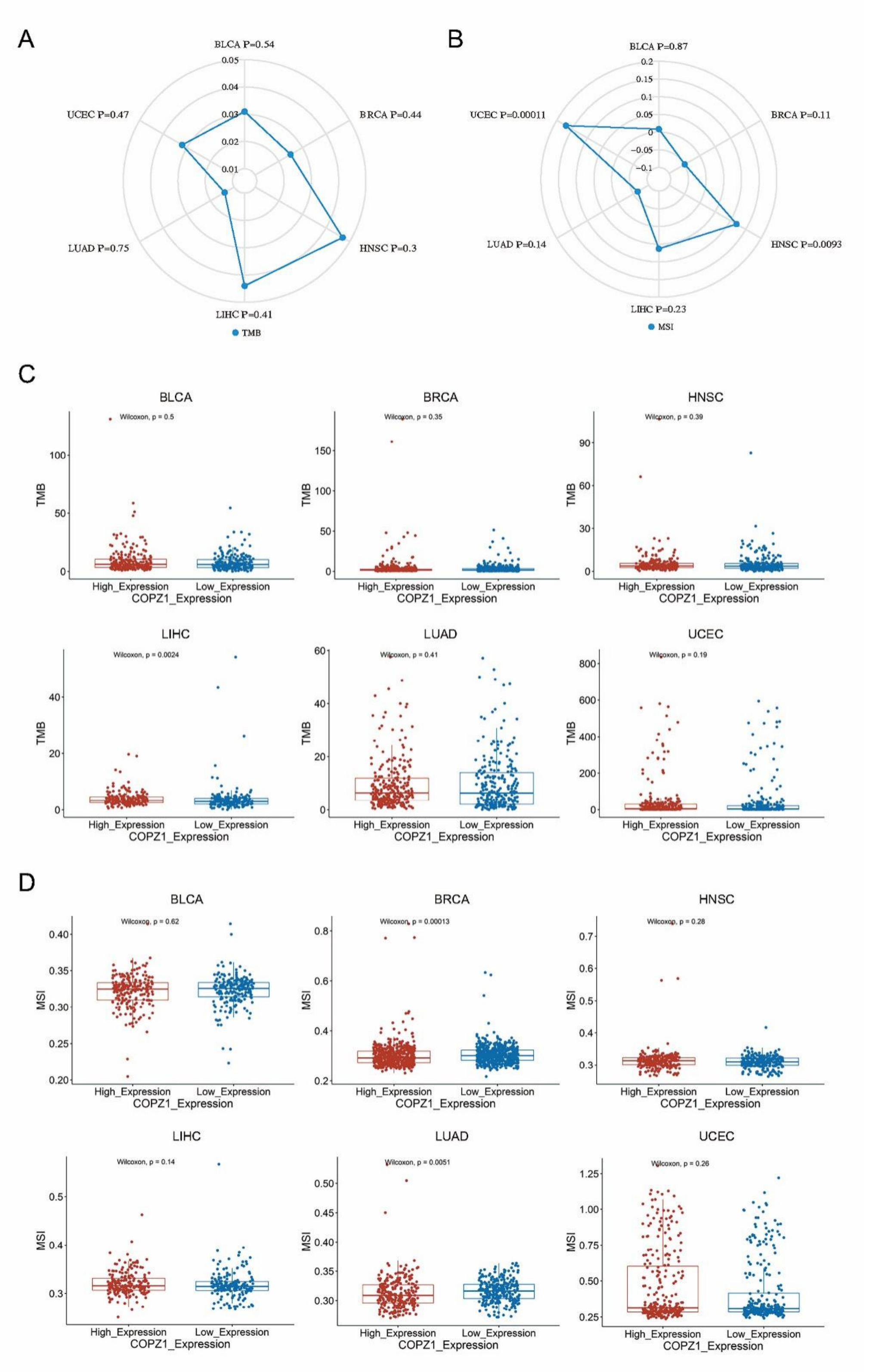

A. Correlation between COPZ1 expression and TMB in multiple cancers. **B**.Correlation between COPZ1 expression and MSI in multiple cancers. **C.** TMB between high and low COPZ1 expression groups. **D**. MSI between high and low COPZ1 expression groups.

Figure S6. KEGG pathway GSEA analysis of COPZ1 high expression in multiple types of cancer.

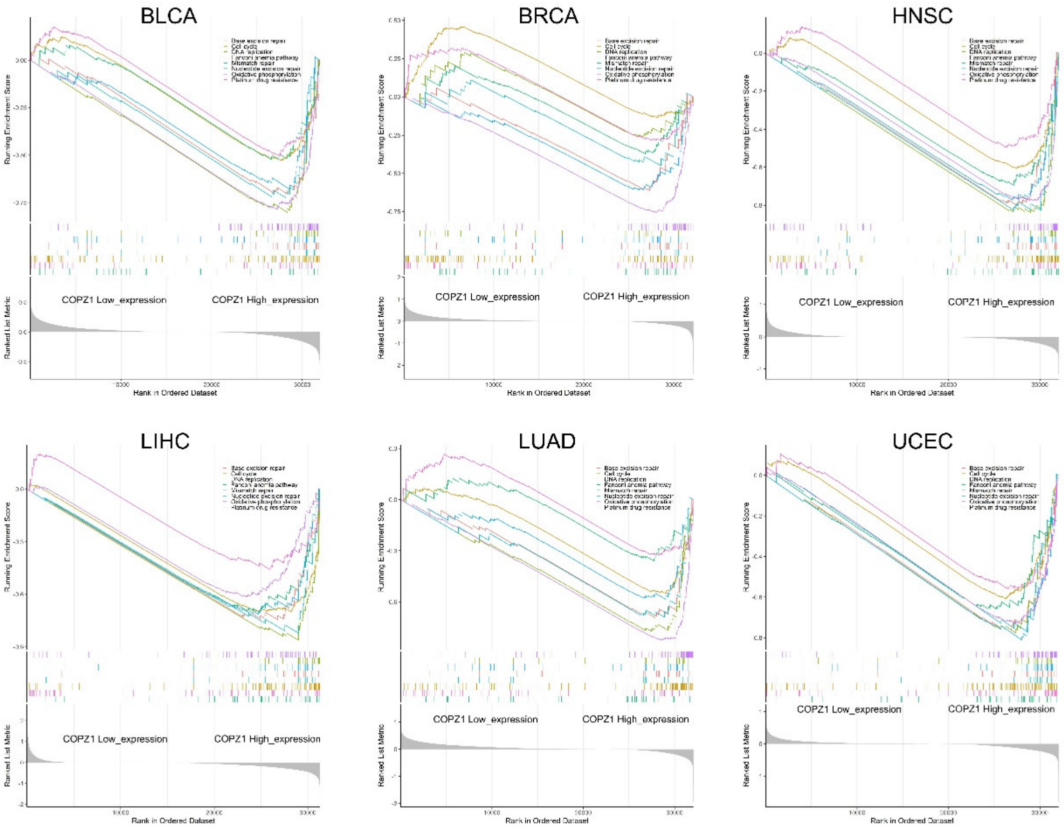

Figure S7. Correlation analysis between COPZ1 expression and immuno-activator expression in multiple cancers.

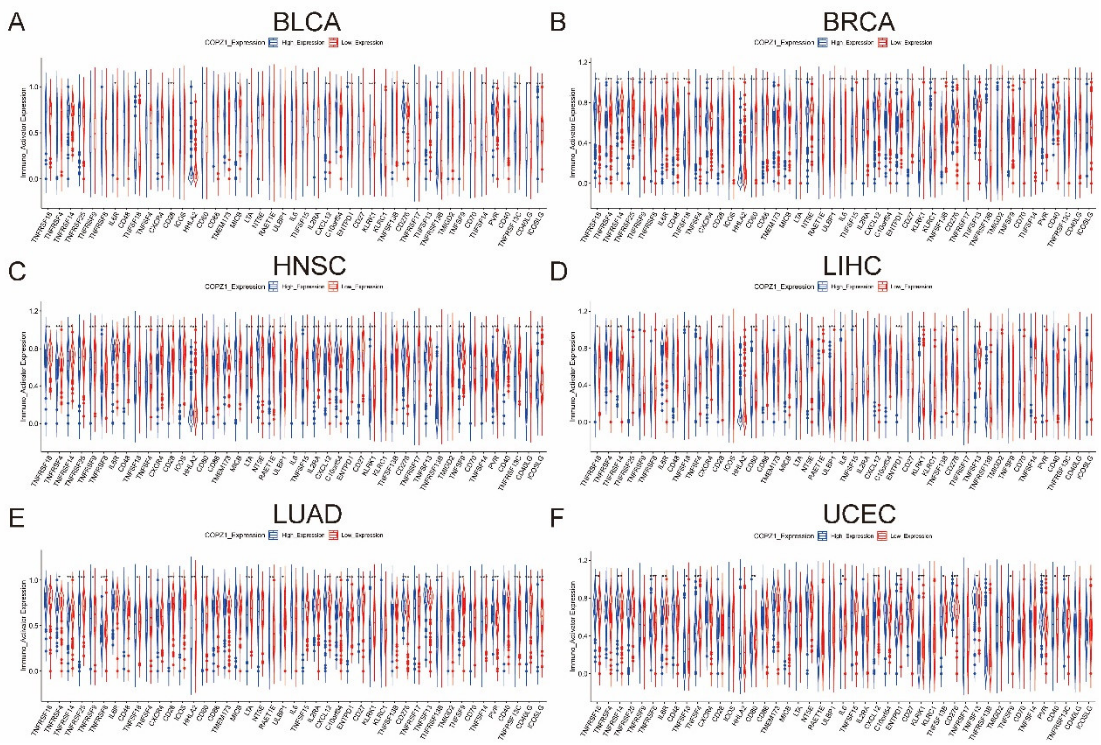

Figure S8. Correlation analysis between COPZ1 expression and immuno-inhibitor expression in multiple cancers.

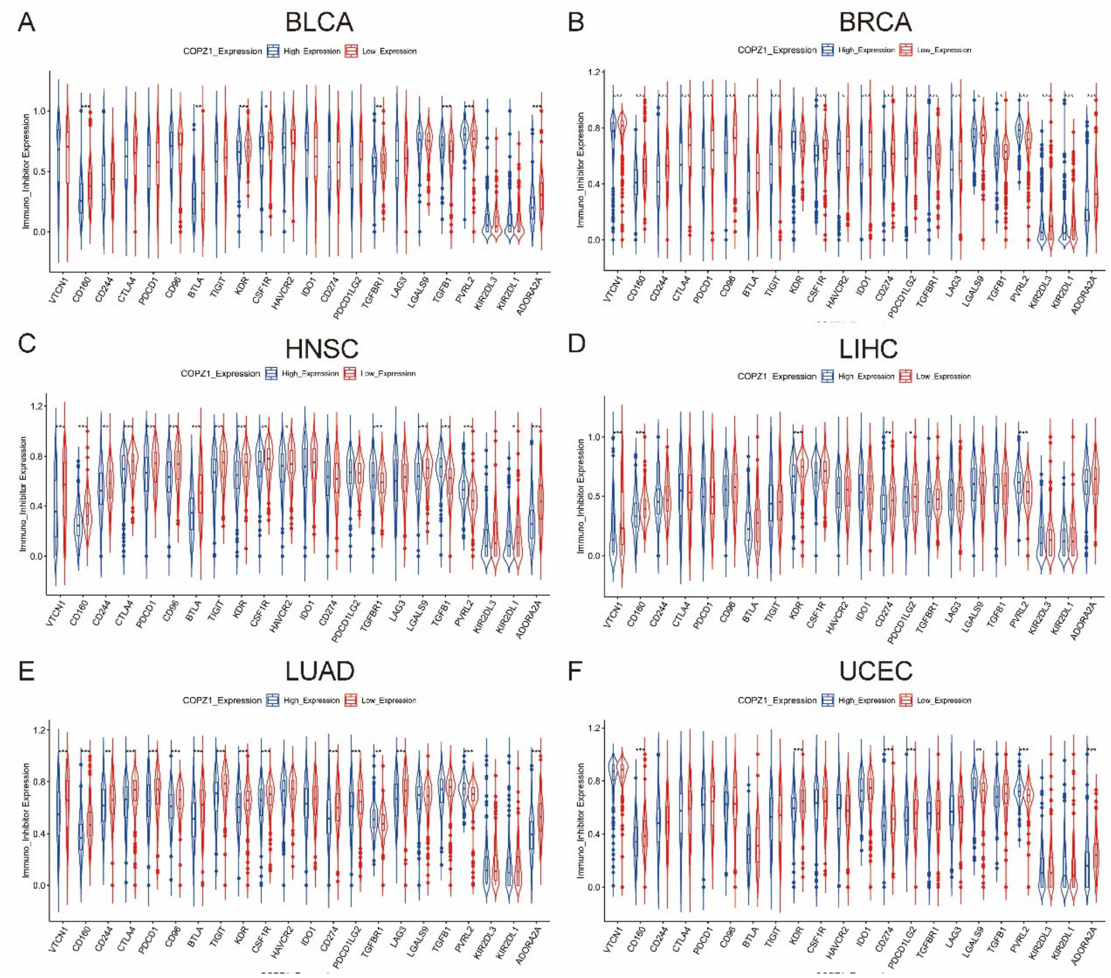

Figure S9. Correlation analysis between COPZ1 expression and cytokine pathway activity

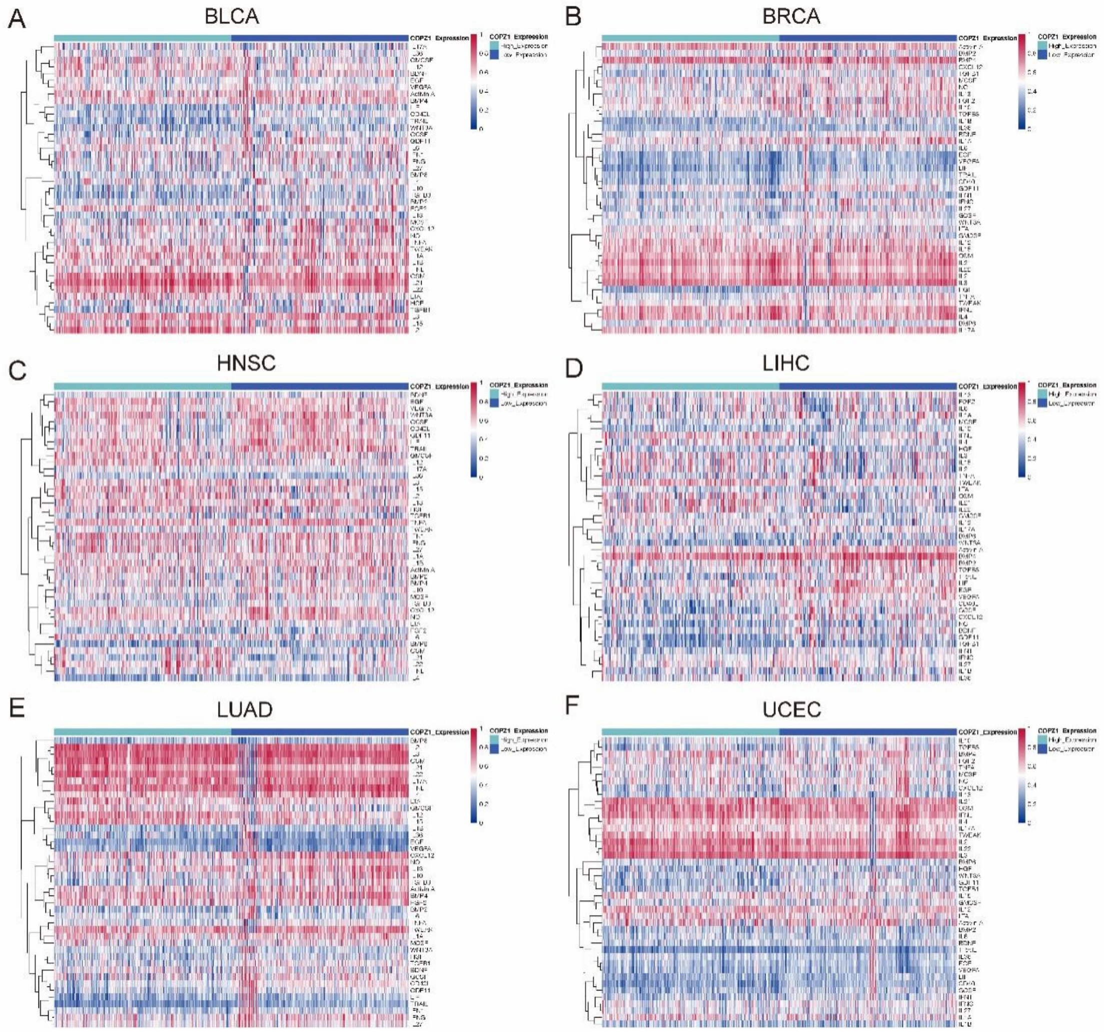

Figure S10. The role of low ZOPC1 expression in LAML and PADC

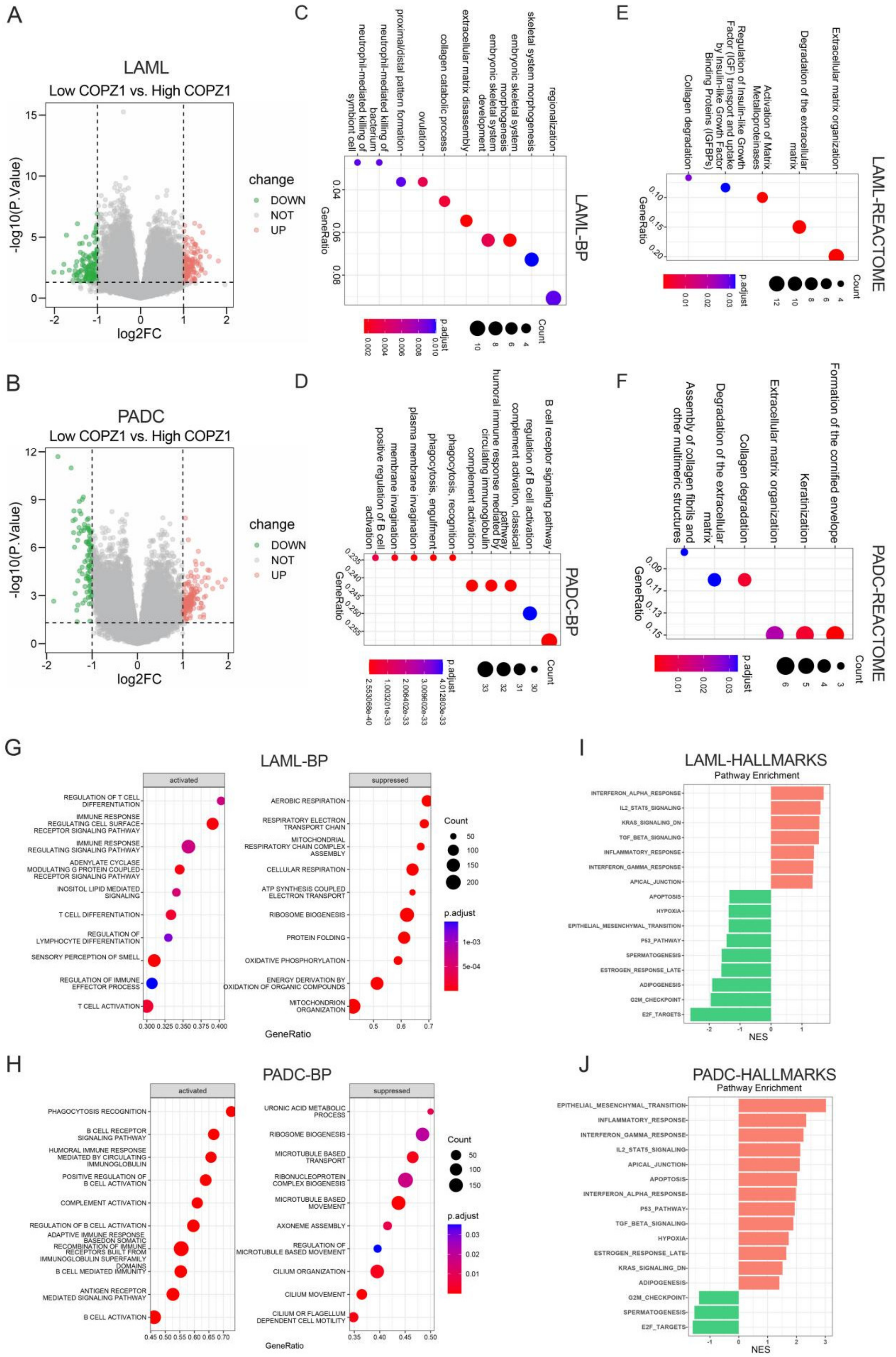

A-B. The volcano plots showed the DGEs in LAML and PADC. **C,E**. The BP and REACTOME enrichment analysis of the DEGs in LAML. **D,F**. The BP and REACTOME enrichment analysis of the DEGs in PADC. **G-H**. Dotplot showed the GSEA enrichment of the BP analysis in the LAML and PADC. **I-J**. The barplot showed the intersected REACTOME pathways enrichment in the LAML and PADC.

Figure S11. The special DEGs and pathways in LAML might regulate the COPZ1

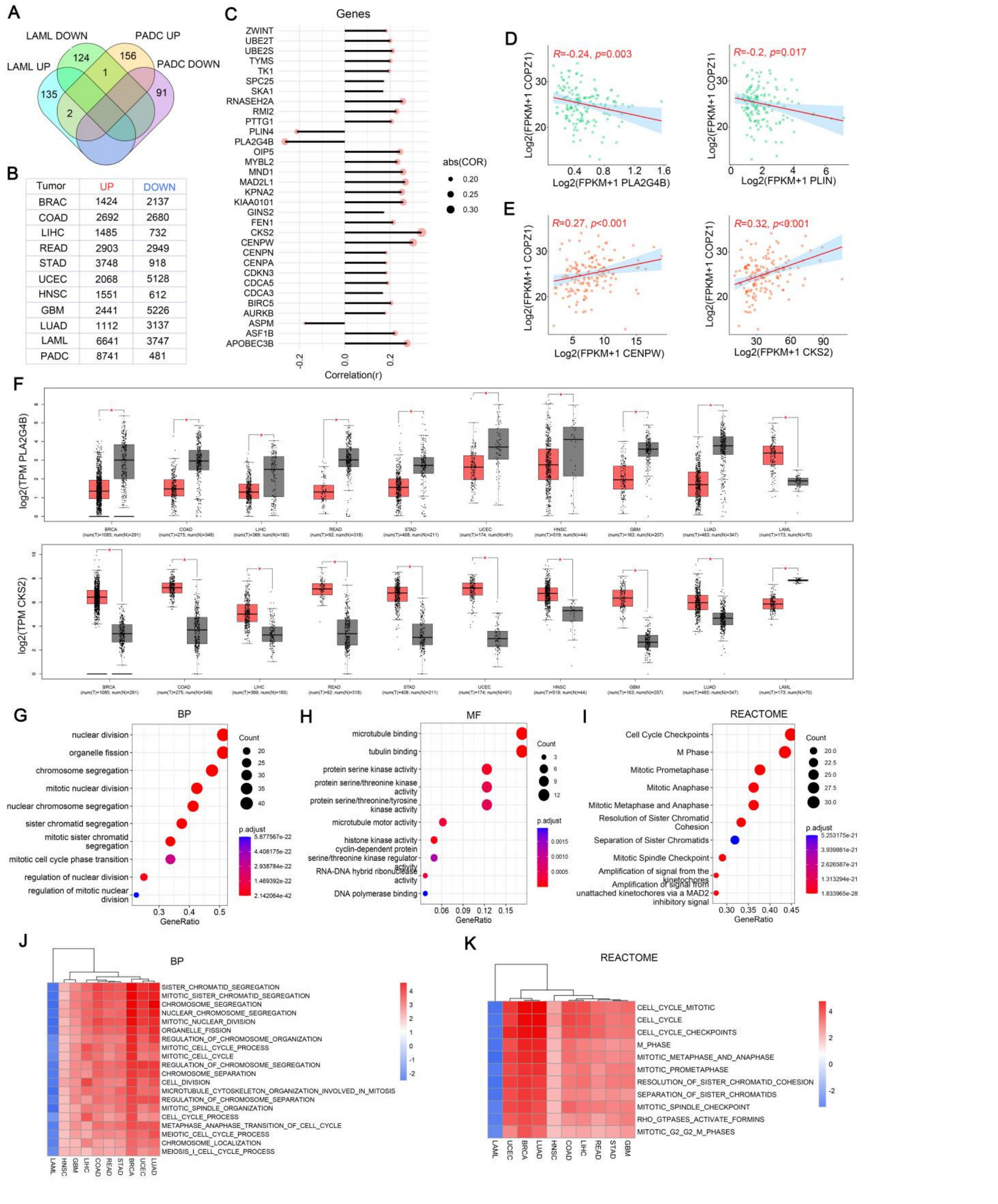

A.The venn plots showed the DEGs between tumor and normal tissue in LAML and PADC. **B**. The DEGs between tumor and normal tissue in 11 types of tumor. **C**. The correlations analysis of the special DEGs in LAML and the COPZ1 expression. **D-E**. The scatter plot showed the top two genes with positive and negative relation with COPZ1 respectively. **F.** The boxplot showed the expression level of PLA2G4B and CKS2 between tumor and normal tissue in 10 types of tumor in GEPIA. **G-I:** The BP, MF and REACTOME analysis of the special DEGs in the LAML. **J-K.** The GSEA of BP and REATOME pathways that suppressed in LAML and activated in the rest 9 types of tumor.
